# Bridging the intergenerational gap: the outcomes of a student-initiated, longitudinal, inter-professional, inter-generational home visit program

**DOI:** 10.1186/s12909-020-02064-x

**Published:** 2020-05-11

**Authors:** Kennedy Yao Yi Ng, Gloria Yao Chi Leung, Angeline Jie-Yin Tey, Jia Quan Chaung, Si Min Lee, Amrish Soundararajan, Ka Shing Yow, Nerice Heng Wen Ngiam, Tang Ching Lau, Sweet Fun Wong, Chek Hooi Wong, Gerald Choon-Huat Koh

**Affiliations:** 1 Department of Medical Oncology, National Cancer Centre Singapore, Singapore, Singapore; 2 Department of Internal Medicine, Changi General Hospital, Singapore, Singapore; 3 Department of Intensive Care Medicine, Sengkang General Hospital, Singapore, Singapore; 4 Changi Naval Base, Singapore Armed Forces, Singapore, Singapore; 5 Department of General Medicine, Tan Tock Seng Hospital, Singapore, Singapore; 6 Tekong Island Medical Centre, Singapore Armed Forces, Singapore, Singapore; 7 Department of General Surgery, Changi General Hospital, Singapore, Singapore; 8 Department of Internal Medicine, Singapore General Hospital, Singapore, Singapore; 9 Department of Medicine, Yong Loo Lin School of Medicine, National University of Singapore, National University Hospital, Singapore, Singapore; 10 Populational Health and Community Transformation, Khoo Teck Puat Hospital, Singapore, Singapore; 11 Health Services and Systems Research, Duke-NUS Medical School, Singapore, Singapore; 12 Department of Geriatric Medicine, Khoo Teck Puat Hospital, Singapore, Singapore; 13 Geriatric Education and Research Institute, Singapore, Singapore; 14 Yong Loo Lin School of Medicine, National University of Singapore, National University Health System, Singapore, 117549 Singapore; 15 Saw Swee Hock School of Public Health, National University of Singapore, Singapore, 117549 Singapore

**Keywords:** Intergeneration interactions, Interprofessional, Community medicine, Ageism

## Abstract

**Background:**

Older persons consume disproportionately more healthcare resources than younger persons. Tri-Generational HomeCare (TriGen), a service-learning program, aims to reduce hospital admission rates amongst older patients with frequent admissions. The authors evaluated the educational and patient outcomes of TriGen.

**Methods:**

Teams consisting of healthcare undergraduates and secondary school (SS) students - performed fortnightly home visits to patients over 6 months. Self-administered scales were used to evaluate the educational outcomes in knowledge and attitudes towards the older people and nine domains of soft skills pre- and post-intervention. Patients’ reported satisfaction and clinical outcomes were also assessed.

**Results:**

Two hundred twenty-six healthcare undergraduates and 359 SS students participated in the program from 2015 to 2018. Response rates were 80.1 and 62.4% respectively. One hundred six patients participated in TriGen. There was a significant increase in Kogan’s Attitudes towards Old People Scale (KOP) scores for healthcare undergraduates and SS students with a mean increase of 12.8 (95%CI: 9.5–16.2, *p* <  0.001) and 8.3 (95%CI: 6.2–10.3, *p* <  0.001) respectively. There was a significant increase in Palmore Facts on Aging Quiz (PFAQ) score for SS students but not for healthcare undergraduates. Most volunteers reported that TriGen was beneficial across all nine domains assessed. There was also a significant decrease in hospital admission rates (*p* = 0.006) and emergency department visits (*p* = 0.004) during the 6-month period before and after the program. Fifty-one patients answered the patient feedback survey. Of this, more than 80% reported feeling less lonely and happier.

**Conclusion:**

TriGen, a student-initiated, longitudinal, inter-generational service-learning program consisting of SS students and healthcare undergraduates can reduce ageism, develop soft skills, inculcate values amongst SS students and healthcare undergraduates. In addition, TriGen potentially reduces hospital admissions and emergency department visits, and loneliness amongst frequently admitted older patients.

## Background

The demographic landscape in Singapore is aging. By 2030, this is estimated to nearly double to 900,000, or 25% of the resident population [1]. This poses many challenges. Even though a relatively small proportion of the population, older persons account for a disproportionate 32.0% of admissions to acute hospitals and 81.0% of admissions to community hospitals [2]. Acute hospitalizations are also associated with complications including nosocomial infections and prolonged hospital stays amongst older adults [3].

Hence, there is increasing interest in avoiding acute hospitalization and promoting community-based healthcare to meet healthcare needs of complex patients [4–6]. The advantages of community-based healthcare include extending the reach of healthcare professionals to those who might otherwise not have sought necessary healthcare [3], by forming a stronger therapeutic relationship between the healthcare professional and the individual [7], and by allowing healthcare professionals to be better able to tackle individuals’ problems holistically and not just medically [8, 9]. If healthcare professionals can identify and address unmet needs in the community, they can potentially prevent avoidable hospital admissions.

Another approach is to reduce loneliness amongst the older persons. It has been shown that loneliness was associated with hospital admissions, increased lengths of stay and overutilization of healthcare resources [10], as well as increased mortality and morbidity [11]. Social inclusion can be a way to reduce this loneliness and prevent social isolation. This can be done by helping older persons develop a diverse social network which includes having meaningful relationships with persons of different age groups [12]. The literature suggests that intergenerational programs can strengthen connections among different age groups, decrease loneliness and social isolation of older person, and in so doing, promote health [13, 14]. In light of the above, we chose to adopt an intergenerational approach in our program by including volunteers of varying age groups – secondary school (SS) students and health undergraduates.

Another proposed intervention is to reduce ageism amongst healthcare undergraduates and the community. It has been shown that ageism amongst healthcare professionals leads to lower quality of care for the older persons [15, 16], possibly due to undertreatment by healthcare professionals [17]. The presence of ageist attitudes amongst healthcare professionals may lead to the older persons feeling powerless and having decreased self-esteem and discourage them from candidly sharing their concerns, preferences and needs [18]. Moreover, according to Jackson, Sarah E et al., perceived age discrimination was associated with increased risk of serious health problems among older persons living in England [19]. Fortunately, studies have also found that ageism amongst healthcare professionals and non-healthcare professionals can be overcome with education on the aging process [20–22] and positive experiences in interacting with the older persons [22–24]. Reducing ageism with these strategies may lead to an increased interest in caring for the older persons [21] and could potentially improve overall quality of care. We opted to target healthcare undergraduates as it is crucial to change the attitudes of healthcare professionals early in their education, since there are studies suggesting that ageism can be amplified as healthcare professionals gain more work experience [25].

Developing empathy, social awareness and other intangible soft skills and values (e.g. teamwork, communication skills, responsibility) amongst the healthcare undergraduates and SS students is also important. It has been shown that empathy level decreases with increasing years of training and education amongst healthcare students and professionals [26–29]. This leads to poorer care and patient outcomes. With greater empathy and social awareness, healthcare undergraduates and SS students will be equipped to provide patient-centered care, which has been shown to reduce healthcare utilization and improve health outcomes [30, 31].

Tri-Generational HomeCare (TriGen) was conceived to reduce unnecessary acute healthcare resources utilization amongst the older persons. It leverages on the aforementioned interventions: 1) community-based care, 2) reducing loneliness amongst the older person through an intergenerational approach, 3) reducing ageism, 4) development of empathy, social awareness and other intangible soft skills and values amongst SS students from the community and healthcare undergraduates. TriGen is based on the service-learning model, with the dual objectives of service to the community and learning by the participants [32, 33], and empowers volunteers (healthcare undergraduates and SS students) to provide holistic care and companionship to at-risk older adults. It also provides a structured learning experience where participants can reflect on and learn from their experiences with the aim of reducing ageism, development of soft skills like empathy, social awareness and inculcating values.

### Program description

TriGen is a collaboration between the National University of Singapore Yong Loo Lin School of Medicine (NUS YLLSoM), Khoo Teck Puat Hospital (KTPH), a regional hospital situated in the Northern part of Singapore, and North West Community Development Council (NWCDC), a social organization based in the northwestern district of Singapore. KTPH developed the aging-in-place (AIP) program that aims to reduce readmission rates amongst high consumers of healthcare, defined as those admitted into hospital three or more times over a six-month period.

TriGen, as the name suggests, involves participants across three generations: i) older patients who are enrolled in the AIP program; ii) healthcare undergraduate enrolled in Medicine, Nursing, Pharmacy, Social Work, Physiotherapy or Occupational Therapy course from various higher educational institutions in Singapore; and iii) SS student, who come mostly from schools in the North-western district.

The learning objectives for healthcare undergraduates and SS students can be summarized in Table 1.

**Table 1:** Learning objectives for TriGen participants and aspects of programmes to meet these objectives

Aims for participants	Aspects of TriGen fulfilling the aims	
	Healthcare undergraduates	Secondary school students
Interprofessional learning	Pre-visitation training ● Healthcare undergraduates completed the pre-visitation training in interprofessional groups During visitations ● Healthcare undergraduates worked in interprofessional groups of two to three undergraduates to conduct home visits During conference sessions ● Multi-disciplinary meetings with healthcare professionals from KTPH (including doctors, nurses, pharmacists and social workers) where students present their assessment of the older persons they have visited and their proposed management plan, with the KTPH faculty will provide additional input and guidance	
Increasing knowledge of the aging process, reducing ageist attitudes, basic caregiving skills	Pre-visitation training ● Lectures on common medical conditions in the older person and the normal aging process ● Lectures and workshop addressing possible difficulties in communicating with older persons and role play on strategies to overcome them ● Role-playing activities to help students understand the sensory deficits and other problems commonly faced by the older persons, and strategies to overcome these problems ● Occupational Therapist/Physiotherapist-led hands-on session exploring mobility aids and skills on transfer of the older persons ● Workshop teaching basic caregiving skills e.g. measurement of blood pressure and capillary blood glucose (for non-medical healthcare undergraduates) During visitations ● Interacting with older persons during home visits, including monitoring of patients’ vital parameters, patient education, medication reconciliation, befriending and social activities, cleaning up of the house, coordination of medical/social services	Pre-visitation training ● Lecture on aging population trends in Singapore ● Workshop addressing possible difficulties in communicating with older persons and role play on strategies to overcome them ● Role-playing activities to help students understand the sensory deficits and other problems commonly faced by the older persons, and strategies to overcome these problems ● Workshop teaching basic caregiving skills e.g. measurement of blood pressure and capillary blood glucose During visitations ● Interacting with older persons during home visits, including monitoring of patients’ vital parameters, patient education, befriending and social activities, cleaning up of the house, under the leadership of the healthcare undergraduates ● Reflections and debrief sessions held after each home visit, facilitated by the healthcare undergraduates, guided by a series of lesson plans ranging from dementia to communication with the older persons
Other knowledge and skills	Pre-visitation training ● Lecture on community resources available to less privileged populations and who to refer them for help ● Lesson on how to provide counseling on lifestyle and diet ● Workshop imparting leadership skills, facilitation skills During visitations ● Leading groups of secondary school students during visitations, facilitating their interactions with older persons ● Facilitating reflections and debrief sessions for the secondary school students after each home visit, guided by a series of lesson plans ● Collaborating with the healthcare professionals from AIP During conference sessions ● Presenting on the issues faced by the older persons they visited to healthcare professionals to update on progress and seek professional input	Pre-visitation training ● Lectures on how to provide counseling on lifestyle and diet During visitations ● Taking vitals under the supervision of the healthcare undergraduates ● Organising of activities to engage the older persons with, e.g. singing, lantern making, gardening ● Reflections sessions held after each home visit, facilitated by the healthcare undergraduates

The program begins with the healthcare undergraduates and SS students undergoing training sessions to prepare them for visitations. Each team comprised two to three undergraduates from different disciplines, and three to five SS students. Each team was allocated one to two patients and fortnightly visits were conducted over 6 months. Twice over these 6 months, after the first few visits and again nearing the conclusion of the visitation period, there will be multi-disciplinary meetings where the healthcare undergraduates present on issues faced by the older patients to healthcare professionals from KTPH, including doctors, pharmacists, nurses and social workers, and sought advice on the most appropriate management for these patients.

### Research aims

In this paper, we aim to evaluate the educational and patient outcomes of a student-initiated, home-based, inter-generational, inter-professional, longitudinal service-learning program focused on older patients who have frequent hospital readmissions. In so doing, we hope that our experiences will be useful in informing existing and future services learning programs of this nature.

## Methods

This study employs a descriptive and a pretest-posttest research design.

### Survey instruments for SS students and healthcare undergraduates

For the volunteers, 3 sets of self-administered scales were used: Kogan’s Attitude towards Old People Scale (KOP) [34] to assess ageist attitudes, Palmore Facts on Aging Quiz (PFAQ) [35] to assess knowledge of older persons, and Fund for the Improvement of Post-Secondary Education Survey Instrument (FIPSE) [36] to assess pedagogical value.

The KOP consists of 17 paired statements on a 5-point Likert scale, one of each pair positively framed and the other negatively framed. Respondents were asked to indicate the level to which they agree or disagree using a 5-point Likert scale. The scores range from 34 to 170 with a neutral score of 102. A higher score indicates a less negative attitude towards older persons. The KOP has been validated by multiple authors in healthcare undergraduates and professionals [37, 38].

The PFAQ consists of 25 true/false statements that query health and socioeconomic information about the elderly in the United States. We adapted it to the local context by replacing the term United States with Singapore in the questionnaire. The scale is scored with 1 point given for every correct answer, with a maximum score of 25. A higher score indicates better knowledge about the older persons. The PFAQ has been validated in different groups of learners [39, 40].

The FIPSE was adapted and comprises nine domains including leadership, communication skills, teamwork, critical thinking skills, ability to identify social issues, actions skills (i.e. the abilities to take action and take on new responsibilities), the ability to see consequences, the acquisition of knowledge, the application of knowledge. This was previously validated in a group of Taiwanese medical students [41], and used in multiple other studies [42, 43]. Respondents were asked to answer the questions using a 5-point Likert scale ranging from strongly agree, agree, neutral, disagree, strongly disagree.

#### Internal consistency of survey instruments

Cronbach’s alpha (α) was calculated to check for internal consistency of the above instruments. For the KOP scale, α = 0.88 for the healthcare undergraduates and 0.75 for the SS students. The scale remained consistent when divided into positively worded statements (α = 0.75 for the healthcare undergraduates and 0.74 for the SS students) and negatively worded statements (α = 0.95 for the healthcare undergraduates and 0.74 for the SS students). For PFAQ, α = 0.49 for the healthcare undergraduates and 0.50 for the SS students. For FIPSE, α = 0.88 for the healthcare undergraduates and 0.98 for the SS students.

### Patients’ reported satisfaction and clinical outcomes

For the patients, a patient feedback survey consisting of quantitative questions and open-ended questions was administered to the patients.

All scales were administered at two time points (pre- and post-intervention) except the FIPSE and patient feedback survey which were only administered post-intervention.

Number of hospital admissions and emergency department visits by the patients under TriGen during the 6-months period before and after the program was collected from the hospital’s administrative database.

### Procedure

We obtained ethical approval from the NUS institutional review board (B-15-272) and KTPH domain specific review board (2015/01220) to evaluate the learning outcomes and the patient outcome of the TriGen respectively. Study participation for both the volunteers and patients was entirely voluntary and anonymous. We took informed consent from both the healthcare undergraduate and the patients and parental consent for the SS students as they were under 21 years-old at the start of the program. Non-participation in the study did not impact students’ evaluations. Patients who did not participate in the study continued to receive care from TriGen. There were no incentives provided to all participants for completing the questionnaires.

### Statistical analysis

Statistical analysis was carried out on the baseline and post-intervention questions for the aforementioned scales. We used the Shapiro-Wilk test to assess if the data follows a normal distribution [44]. If the data follows a normal distribution, we used parametric tests. Otherwise, we employed non-parametric tests. A paired *t*-test comparing baseline and post-intervention responses was computed for each survey item to determine significant attitude differences (*p* ≤ 0.05). One-way ANOVA was performed to assess for demographic factors that correlated with pre-intervention and magnitude of change in KOP, PFAQ scores. If one-way ANOVA demonstrated an overall difference between groups, we proceeded to perform a post-hoc Tukey’s honestly significant difference (Tukey’s HSD) test. This post-hoc test controls for the familywise Type 1 error and provide adequate statistical power [45]. We also computed descriptive statistics for the FIPSE and used chi-square analysis to compare the self-reported learning in the nine domains between genders, between pre-clinical (first- and second-year) and clinical (third- to fifth-year) students. We used logistic regression to adjust for clinical exposure when comparing learning between genders amongst healthcare undergraduates and to adjust for age when comparing learning between genders amongst SS students. The Wilcoxon signed-rank test was used to evaluate the difference between the number of emergency department visits and hospital admissions in the 6 months period before and after the program.

For all statistical analyses, we used Statistical Package for Social Sciences (SPSS, Version 23.0, Chicago, Illinois).

## Results

There is a total of 226 healthcare undergraduates and 359 SS students who participated in the program from 2015 to 2018. Response rate was 80.1 and 62.4% amongst the healthcare undergraduates and SS students respectively. Table 2 describes the profile of the participants.

**Table 2:** Demographic profile of students who participated in the TriGen Programme from 2014 to 2017

	Healthcare undergraduates	Secondary school students
Total number of participants	226	359
Number of respondents	181	224
Response rate	80.1%	62.4%
Median Age (Range)	21 (18–41)	15 (13–17)
**Gender**		
Male	68 (37.6%)	79 (35.3%)
Female	113 (62.4%)	145 (64.7%)
**Faculty/School**		
	Medicine: 57 (31.5%)	West Spring Secondary School: 80 (35.7%)
	Nursing: 23 (12.7%)	Yishun Secondary School: 112 (50%)
	Pharmacy: 76 (42.0%)	Orchid Park Secondary School: 25 (11.2%)
	Social Work: 20 (11.0%)	Chung Cheng High School: 1 (0.4%)
	Physiotherapy and Occupational Therapy: 5 (2.8%)	Anglo-Chinese School (Independent): 3 (1.3%)
		Eunoia Junior College: 3 (1.3%)
**Year of study**		
Year 1	72 (39.8%)	–
Year 2	41 (22.7%)	37 (16.5%)
Year 3	52 (28.7%)	178 (79.5%)
Year 4	14 (7.7%)	3 (1.3%)
Year 5	2 (1.7%)	6 (2.7%)
**Living with grandparents**		
Yes	25 (13.8%)	49 (21.9%)
No	156 (86.2%)	175 (78.1%)
**Involved in volunteer work with the older person**		
Yes	131 (72.4%)	123 (54.9%)
No	50 (27.6%)	101 (45.1%)
**Hours spent on CIP**		
**Excluding training**		410 mins
**Inclusive of training**		480 mins
**Previous IPE activities**		
	Yes: 117 (64.6%)	
	No: 64 (35.4%)	

### Kogan’s attitude towards old people scale

#### Healthcare undergraduates

There was a statistically significant increase in KOP score pre- and post-intervention for healthcare undergraduates with a mean increase of 12.8 (95% CI: 9.46–16.2, *p* <  0.001). (Table 3) This increase was found in all groups of undergraduates. All subgroups analysed had a statistically significant increase in KOP score pre- and post-intervention. There was a statistically significant difference in pre-intervention KOP score between those who stay with their grandparents and those who do not (*p* = 0.03). A Tukey post hoc test revealed that undergraduates who stay with their grandparents have higher pre-intervention KOP scores (i.e. have less ageist attitudes) (mean score 138.3, 95% CI: 132.5–144.2) as compared to those who do not (mean score 132.1, 95% CI: 128.9–135.3). There was no statistically significant difference for pre-intervention KOP found for different genders (male versus female), seniority (preclinical versus clinical), previous or current volunteer work involving the older persons.

**Table 3:** KOP score in university healthcare undergraduates

Group	No.	Pre-Intervention Score (mean, 95%CI)	***P***-value (comparison between groups for pre-intervention score)	Post-Intervention Score (mean, 95%CI)	Mean Difference	***P***-value	Comparison between groups
**All University Students**	172	133.0 (130.1–135.9)		145.7 (143.3–148.1)	12.8 (9.4–16.2)	**< 0.0001**	
**Gender**			0.26				0.075
Male	64	135.5 (131.2–139.8)		144.3 (139.6–148.9)	8.8 (3.6–14.0)	**0.001**	
Female	108	131.5 (127.6–135.3)		146.6 (143.9–149.3)	15.1 (10.7–19.6)	**< 0.0001**	
**Year of Study**			0.15				0.43
Year 1 and 2	108	134.8 (130.6–138.9)		146.5 (143.3–149.7)	11.7 (7.1–16.4)	**< 0.0001**	
Year 3 to 5	64	130.0 (126.7–133.2)		144.5 (140.9–148.1)	14.5 (9.7–19.3)	**< 0.0001**	
**Faculty**			0.78				0.53
Medical	57	132.8 (126.1–139.5)		148.9 (144.9–153.0)	16.1 (8.4–23.9)	**< 0.0001**	
Nursing	23	134.2 (128.8–139.7)		147.7 (142.9–152.4)	13.4 (8.3–18.6)	**< 0.0001**	
Pharmacy	68	132.5 (128.5–136.6)		141.7 (137.3–146.1)	9.18 (4.00–14.36)	**0.001**	
Social Work	19	131.8 (125.5–138.1)		146.6 (141.1–152.1)	14.79 (7.63–21.95)	**< 0.0001**	
Therapist	5	139.4 (133.0–145.8)		151.6 (142.7–160.5)	12.2 (3.0–21.4)	**0.021**	
**Living with grandparents**			**0.032**				0.65
Yes	24	138.3 (132.5–144.2)		149.1 (143.2–155.1)	10.8 (2.73–18.9)	**0.011**	
No	148	132.1 (128.9–135.3)		145.2 (142.5–147.8)	13.1 (9.3–16.8)	**< 0.0001**	
**Have you volunteered in an old person facility?**			0.97				0.13
Yes	127	132.9 (129.2–136.5)		147.2 (144.2–150.1)	14.3 (9.9–18.7)	**< 0.0001**	
No	45	133.2 (129.0–137.4)		141.6 (137.7–145.6)	8.4 (4.5–12.3)	**< 0.0001**	

Pre-intervention KOP scores is weakly positively associated with post-intervention KOP scores (*r* = 0.177, *p* = 0.020) and moderately negatively associated with the difference in KOP scores (*r* = − 0.724, *p* <  0.001).

#### Secondary school students

There was a statistically significant increase in KOP score pre- and post-intervention for SS students with a mean increase of 8.3 (95% CI: 6.2–10.3, *p* <  0.001). (Table 4) All subgroups analysed had a statistically significant increase in KOP score pre- and post-intervention. There was no statistically significant difference for pre-intervention KOP found between the different genders (male versus female), secondary schools, seniority (lower secondary versus upper secondary), whether they are living with their grandparents or having previous volunteering experience in an old person facility.

**Table 4:** KOP score in secondary school students

Group	No.	Pre-Intervention Score (mean, 95%CI)	***P***-value (comparison between groups for pre-intervention score)	Post-Intervention Score (mean, 95%CI)	Mean Difference	***P***-value	Comparison between groups
**All secondary school**	224	127.4 (125.6–129.2)		135.7 (134.1–137.3)	8.3 (6.2–10.3)	**< 0.001**	
**Gender**			0.27				0.55
Male	79	128.78 (125.21–132.36)		136.19 (133.68–138.71)	7.41 (3.46–11.35)	**< 0.001**	
Female	145	126.67 (14.66–128.67)		135.42 (133.07–137.77)	8.75 (6.43–11.07)	**< 0.001**	
**Age**			0.12				0.69
Younger (Age 13–14)	63	125.16 (122.69–127.63)		134.05 (130.47–137.63)	8.89 (5.18–12.59)	**< 0.001**	
Older (Age 15–17)	161	128.30 (125.98–130.61)		136.34 (134.33–138.34)	8.04 (5.59–10.48)	**< 0.001**	
**School**			0.43				0.54
School 1 (Westspring)	80	127.48 (124.31–130.64)		137.84 (135.24–140.44)	10.36 (6.83–13.90)	**< 0.001**	
School 2 (Yishun Sec)	112	126.30 (123.83–128.76)		133.82 (131.11–136.53)	7.53 (4.49–10.57)	**< 0.001**	
Others	32	131.10 (126.47–135.91)		136.88 (132.71–141.04)	5.69 (2.01–9.36)	**0.004**	
**Living with grandparents**			0.45				0.84
Yes	49	126.12 (122.70–129.54)		133.96 (131.02–136.90)	7.84 (4.36–11.32)	**< 0.001**	
No	175	127.78 (125.69–129.86)		136.18 (134.08–138.27)	8.40 (5.98–10.82)	**< 0.001**	
**Have you volunteered in an old person facility?**			0.37				0.26
Yes	123	128.15 (125.94–130.37)		135.41 (132.79–138.02)	7.25 (4.56–9.94)	**< 0.001**	
No	101	126.52 (123.59–129.44)		136.04 (133.78–138.30)	9.52 (6.40–12.65)	**< 0.001**	
**Do you have any siblings?**			0.56				0.36
Yes	185	127.17 (125.18–129.17)		135.89 (133.98–137.81)	8.72 (6.41–11.03)	**< 0.001**	
No	39	128.56 (124.52–132.61)		134.74 (130.31–139.18)	6.18 (2.11–10.25)	**0.004**	

Number of hours spent on home visits is weakly positively associated with difference in KOP scores (*r* = 0.234, *p* <  0.001). Pre-intervention KOP scores is weakly positively associated with post-intervention KOP scores (*r* = 0.333, *p* <  0.001) and moderately negatively associated with the difference in KOP scores (*r* = − 0.598, *p* <  0.001).

The baseline KOP score of the healthcare undergraduates is significantly higher than that of the secondary school students (mean = 6.8, 95% CI: 3.7 to 9.9, *p* <  0.001), and the increase in KOP score for healthcare undergraduates was also significantly more than that of the secondary school students (mean = 4.6, 95% CI: 0.7 to 8.4, *p* = 0.022).

### Palmore’s facts of aging quiz

#### Healthcare undergraduates

The average pre- and post-intervention PFAQ score is 15.8 and 16.0 respectively, but there was no significant difference in PFAQ score (*p* = 0.112) (Table 5).

**Table 5:** Palmore score in healthcare undergraduate

Group	No.	Pre-Intervention Score (mean, 95%CI)	***P***-value (comparison between groups for pre-intervention score)	Post-Intervention Score (mean, 95%CI)	Mean Difference	***P***-value	Comparison between groups
**All university students**	127	15.7 (15.1–16.2)		16.2 (15.7–16.6)	0.49 (− 0.095–1.07)	0.10	
**Gender**			**0.050**				0.43
Male	48	16.3 (15.5–17.1)		16.5 (15.9–17.1)	0.19 (− 0.61–0.98)	0.64	
Female	79	15.3 (14.5–16.1)		15.9 (15.4–16.5)	0.67 (− 0.14–1.49)	0.11	
**Year of Study**			0.13				0.087
Year 1 and 2	93	16.0 (15.4–16.7)		16.2 (15.8–16.7)	0.18 (− 0.48–0.85)	0.59	
Year 3 to 5	34	14.7 (13.5–15.8)		16.0 (15.1–16.8)	1.32 (0.11–2.54)	**0.034**	
**Faculty**			0.20				0.14
Medical	44	16.0 (14.9–17.1)		16.8 (16.2–17.4)	0.77 (−0.33–1.87)	0.16	
Nursing	14	13.8 (11.2–16.3)		15.5 (14.4–16.6)	1.71 (− 1.05–4.48)	0.20	
Pharmacy	51	16.1 (15.3–16.8)		15.7 (15.0–16.4)	- 0.37 (− 1.11–0.36)	0.31	
Social Work	13	15.2 (13.7–16.7)		16.7 (15.4–18.0)	1.54 (− 0.16–3.24)	0.072	
Therapist	5	15.0 (13.6–16.4)		15.6 (13.9–17.3)	0.60 (− 1.98–3.18)	0.553	
**Living with grandparents**			0.14				0.55
Yes	18	16.3 (15.3–17.2)		16.3 (15.2–17.5)	0.056 (− 1.12–1.23)	0.922	
No	109	15.6 (14.9–16.2)		16.1 (15.7–16.6)	0.56 (− 0.097–1.22)	0.094	
**Have you volunteered in an old person facility?**			0.59				0.26
Yes	95	15.8 (15.1–16.5)		16.1 (15.7–16.6)	0.30 (− 0.43–1.02)	0.42	
No	32	15.1 (14.1–16.2)		16.2 (15.3–17.1)	1.06 (0.17–1.96)	**0.021**	

There is a weak positive correlation between: i) baseline KOP and baseline PFAQ scores (*ρ* = 0.183, *p* = 0.008); ii) post-intervention KOP and post-intervention PFAQ scores (ρ = 0.373, *p* <  0.001); and iii) change in KOP and change in PFAQ scores (*ρ* = 0.266, *p* <  0.001).

#### Secondary school students

There was a statistically significant increase in PFAQ score pre- and post-intervention for SS students with a mean increase of 0.8 (95% CI: 0.3–1.4, *p* = 0.005). (Table 6) Female students, older students, those who do not live with their grandparents, those who had never volunteered in an old person facility and those who have siblings were associated with a statistically significant increase in PFAQ score. There was no statistically significant difference in pre-intervention PFAQ score between the different genders (male versus female), secondary schools, seniority (lower secondary versus upper secondary), whether they are living with their grandparents or having previous volunteering experience in an old person facility.

**Table 6:** Palmore score in secondary school students

Group	No.	Pre-Intervention Score (mean, 95%CI)	***P***-value (comparison between groups for pre-intervention score)	Post-Intervention Score (mean, 95%CI)	Mean Difference	***P***-value	Comparison between groups for mean difference
**All secondary school**	162	13.3 (12.9–13.8)		14.2 (13.6–14.7)	0.81 (0.25–1.38)	**0.005**	
**Gender**			0.061				0.57
Male	64	13.89 (13.17–14.61)		14.53 (13.79–15.28)	0.64 (−0.12–1.40)	0.097	
Female	98	12.99 (12.38–13.60)		13.92 (13.13–14.71)	0.93 (0.13–1.73)	**0.023**	
**Age**			0.15				0.21
Younger (Age 13–14)	32	14.03 (12.89–15.17)		14.16 (12.83–15.48)	0.13 (−1.18–1.43)	0.85	
Older (Age 15–17)	130	13.18 (12.67–13.69)		14.16 (13.54–14.78)	0.98 (0.36–1.61)	**0.002**	
**School**			0.23				0.15
School 1 (Westspring)	52	13.77 (12.92–14.62)		14.08 (13.18–14.97)	0.31 (− 0.66–1.28)	0.53	
School 2 (Yishun Sec)	81	12.90 (12.23–13.57)		13.90 (13.09–14.71)	1.00 (0.23–1.77)	**0.011**	
Others	29	13.82 (12.82–14.84)		15.03 (13.57–16.50)	1.21 (−0.44–2.85)	0.14	
**Living with grandparents**			0.75				0.22
Yes	37	13.49 (12.66–14.31)		13.68 (12.52–14.84)	0.19 (− 0.93–1.31)	0.73	
No	125	13.30 (12.75–13.86)		14.30 (13.66–14.95)	1.00 (0.35–1.65)	**0.003**	
**Have you volunteered in an old person facility?**			0.66				0.49
Yes	88	13.25 (12.64–13.86)		13.86 (13.10–14.63)	0.61 (−0.11–1.33)	0.093	
No	74	13.56 (12.73–14.19)		14.51 (13.69–15.34)	1.05 (0.15–1.96)	**0.023**	
**Do you have any siblings?**			0.084				0.58
Yes	128	13.56 (13.02–14.09)		14.28 (13.66–14.90)	0.73 (0.11–1.34)	**0.021**	
No	34	12.56 (11.67–13.45)		13.71 (12.41–15.00)	1.15 (−0.27–2.57)	0.11	

There is no correlation between time spent visiting the elderly and change in PFAQ scores (*ρ* = 0.136, *p* = 0.084). There is a moderate positive correlation between: i) baseline KOP and baseline PFAQ scores (*ρ* = 0.437, *p* <  0.001); and ii) post-intervention KOP and post-intervention PFAQ scores (*ρ* = 0.472, *p* <  0.001). There is a weak positive correlation between the change in KOP scores and the change in PFAQ scores (*ρ* = 0.349, *p* <  0.001).

Healthcare undergraduates had a significantly higher baseline (2.5, 95% CI: 1.8 to 3.1, *p* <  0.001) and post-intervention (1.8, 95% CI: 1.1 to 2.5, *p* <  0.001) PFAQ score than SS students. However, the healthcare undergraduates did not have a significant difference in change in PFAQ score compared to the SS students (− 0.3, 95% CI: − 1.1 to 0.5, *p* = 0.412).

### Fund for improving postsecondary education survey

#### Healthcare undergraduates

Most healthcare undergraduates felt that TriGen was beneficial across all nine FIPSE domains (Table 7). 90–100% of students reported learning in all 9 domains except for ability to make clinical diagnosis (81.6%) and apply what they have learnt in the training sessions to the home visits (80.1%).

**Table 7:** FIPSE in healthcare undergraduates

Domains of learning—“I feel that TriGen has helped me”	Total no. (%), ***n*** = 196	Male students, no. (%), ***n*** = 68	Female students, no.(%), ***n*** = 122	Males versus females			Year 1 and 2, no. (%), ***n*** = 119	Year 3 and above, no. (%), ***n*** = 71	Pre-clinical versus Clinical		
				Pearson Chi-Square	Unadjusted odds ratio (OR) (95% confidence interval)	***P*** value			Pearson Chi-Square	Unadjusted odds ratio (OR) (95% confidence interval)	***P*** value
***Leadership Skills***											
Feel responsible for others in the Community	191 (97.4)	65 (95.6)	120 (98.4)	1.31	0.361 (0.059–2.22)	0.25	116 (97.5)	69 (97.2)	0.015	1.12 (0.183–6.88)	0.90
Improve my leadership skills	188 (95.9)	63 (92.6)	119 (97.5)	2.59	0.318 (0.074–1.37)	0.11	115 (96.6)	67 (94.4)	0.569	1.72 (0.416–7.09)	0.45
***Communication Skills***											
Participate in community affairs	186 (94.9)	**61 (89.7)**	**119 (97.5)**	**5.38**	**0.22 (0.055–0.88)**	**0.020**^+^	112 (94.1)	68 (95.8)	0.245	0.706 (0.177–2.822)	0.62
Develop communication, listening and negotiation skills	194 (99.0)	67 (98.5)	121 (99.2)	0.178	0.554 (0.034–9.00)	0.67	119 (100)	69 (97.2)	3.39	1.03 (0.989–1.07)	0.066
***Teamwork***											
Think of others	192 (98.0)	65 (95.6)	121 (99.2)	2.734	0.179 (0.018–1.76)	0.098	117 (98.3)	69 (97.2)	0.279	1.696 (0.234–12.31)	0.60
Appreciate teamwork and cooperation among peers	194 (99.0)	67 (98.5)	121 (99.2)	0.178	0.554 (0.034–9.00)	0.67	118 (99.2)	70 (98.6)	0.138	1.69 (0.104–27.4)	0.71
Be tolerant of different people	194 (99.0)	67 (98.5)	121 (99.2)	0.178	0.554 (0.034–9.00)	0.67	118 (99.2)	70 (98.6)	0.138	1.69 (0.104–27.4)	0.71
Respect different opinions	194 (99.0)	67 (98.5)	121 (99.2)	0.178	0.554 (0.034–9.00)	0.67	119 (100)	69 (97.2)	3.39	1.03 (0.989–1.07)	0.066
Compromise	189 (96.4)	64 (94.1)	119 (97.5)	1.44	0.403 (0.088–1.86)	0.23	115 (96.6)	68 (95.8)	0.094	1.29 (0.276–5.84)	0.76
Comprehend the moral and ethical issues in health care	183 (93.4)	63 (92.6)	114 (93.4)	0.043	0.884 (0.277–2.82)	0.84	**107 (89.9)**	**70 (98.6)**	**5.25**	**0.127 (0.016–1.00)**	0.022*
***Ability to see consequences***											
Think about the future	187 (95.4)	62 (91.2)	119 (97.5)	3.92	0.261 (0.063–1.08)	0.048	114 (95.8)	67 (94.4)	0.202	1.36 (0.353–5.25)	0.65
***Critical thinking skills***											
Think critically	186 (94.9)	62 (91.2)	118 (96.7)	2.69	0.350 (0.095–1.29)	0.10	114 (95.8)	66 (93.0)	0.72	1.73 (0.482–6.19)	0.40
***Ability to identify social issues***											
Identify social issues and concerns	192 (98.0)	66 (97.1)	120 (98.4)	0.359	0.55 (0.076–4.00)	0.55	116 (97.5)	70 (98.6)	0.267	0.552 (0.056–5.41)	0.61
***Action skills***											
Take action	186 (94.9)_	**61 (89.7)**	**119 (97.5)**	**5.38**	**0.22 (0.055–0.88)**	**0.020**^+^	113 (95.0)	67 (94.4)	0.031	1.12 (0.306–4.13)	0.86
Build confidence & take on new responsibilities	191 (97.4)	**64 (94.1)**	**121 (99.2)**	**4.37**	**0.132 (0.014–1.21)**	**0.037**	117 (98.3)	68 (95.8)	1.12	2.58 (0.421–15.8)	0.29
***Gaining of knowledge***											
Appreciate and identify gaps or deficiencies in the healthcare system	185 (94.4)	**61 (89.7)**	**118 (96.7)**	**3.94**	**0.295 (0.083–1.05)**	**0.047**	111 (93.3)	68 (95.8)	0.508	0.612 (0.157–2.39)	0.48
Appreciate my own health, living condition	192 (98.0)	66 (97.1)	120 (98.4)	0.359	0.550 (0.076–4.00)	0.55	117 (98.3)	69 (97.2)	0.279	1.70 (0.234–12.3)	0.60
Improve my general knowledge about healthcare	180 (91.8)	61 (89.7)	113 (92.6)	0.482	0.694 (0.246–2.00)	0.49	108 (90.8)	66 (93.0)	0.279	0.744 (0.247–2.24)	0.60
Enhance my understanding of the use of public health measures in resource poor setting	189 (96.4)	64 (94.1)	119 (97.5)	1.44	0.40 (0.088–1.86)	0.23	114 (95.8)	69 (97.2)	0.24	0.661 (0.125–3.50)	0.62
***Application of knowledge***											
Improve my clinical diagnostic skills	160 (81.6)	54 (79.4)	100 (82.0)	0.186	0.849 (0.402–1.79)	0.67	**91 (76.5)**	**63 (88.7)**	**4.35**	**0.413 (0.177–0.964)**	0.037*
Apply what I learnt in the training sessions (the one organized before the start of the home visits)	157 (80.1)	57 (83.8)	94 (77.0)	1.23	1.54 (0.714–3.34)	0.27	**87 (73.1)**	**64 (90.1)**	**7.91**	**0.297 (0.123–0.716)**	0.005*

^+^Significant when adjusted for clinical exposure

*Significant when adjusted for gender

When adjusted for clinical experience, female students were more likely to report gains in the area of participation in community affairs and taking action. When adjusted for gender, students in the clinical phase of their training were more likely to report gains in their clinical diagnostic skills and application of knowledge and skills learned during the training session and the ability to comprehend the moral and ethical issues in healthcare.

#### Secondary school students

Most SS students felt that TriGen was beneficial across all nine FIPSE domains (Table 8). When adjusted for age, females were more likely to report gains in respect different opinion, compromise. When adjusted for gender, older students were more likely to report gains in appreciate teamwork and cooperation among peers, appreciate and identify gaps or deficiency in the healthcare system and enhance understanding of use of public health measures in resource poor setting.

**Table 8:** FIPSE in secondary school students

Domains of learning—“I feel that TriGen has helped me”	Total no. (%)	Female students, no.(%), ***n*** = 98	Male students, no. (%), ***n*** = 65	Males versus females			14 and younger, no. (%), ***n*** = 28	15 and older, no. (%), ***n*** = 135	15 and older versus 14 and younger		
				Pearson Chi-Square	Unadjusted odds ratio (OR) (95% confidence interval)	***P*** value			Pearson Chi-Square	Unadjusted odds ratio (OR) (95% confidence interval)	***P*** value
***Leadership Skills***											
Feel responsible for others in the Community	155 (95.1)	94 (95.9)	61 (93.8)	0.36	0.649 (0.156–2.692)	0.55	27 (96.4)	128 (94.8)	0.129	0.677 (0.080–5.733)	0.72
Improve my leadership skills	136 (83.4)	81 (82.7)	55 (84.6)	0.109	1.154 (0.492–2.708)	0.74	23 (82.1)	113 (83.7)	0.041	1.117 (0.383–3.254)	0.84
***Communication Skills***											
Participate in community affairs	145 (89.0)	86 (87.8)	59 (90.8)	0.361	1.372 (0.488–3.861)	0.55	23 (82.1)	122 (90.4)	1.598	2.040 (0.663–6.275)	0.21
Develop communication, listening and negotiation skills	155 (95.1)	95 (96.9)	60 (92.3)	1.796	0.379 (0.087–1.644)	0.18	25 (89.3)	130 (96.3)	2.442	3.120 (0.700–13.899)	0.12
***Teamwork***											
Think of others	156 (95.7)	95 (96.9)	61 (93.8)	0.909	0.482 (0.104–2.226)	0.34	27 (96.4)	129 (95.6)	0.043	0.796 (0.092–6.886)	0.84
Appreciate teamwork and cooperation among peers	158 (96.9)	95 (96.9)	63 (96.9)	0.001	0.995 (0.162–6.123)	1.0	25 (89.3)	133 (98.5)	6.648	7.980 (1.268–50.221)	0.01*
Be tolerant of different people	159 (97.5)	95 (96.9)	64 (98.5)	0.379	2.021 (0.206–19.863)	0.54	27 (96.4)	132 (97.8)	0.176	1.630 (0.163–16.266)	0.68
Respect different opinions	156 (95.7)	97 (99.0)	59 (90.8)	6.41	0.101 (0.012–0.863)	**0.011**^+^	27 (96.4)	129 (95.6)	0.043	0.796 (0.092–6.886)	0.84
Compromise	151 (92.6)	95 (96.9)	56 (86.2)	6.665	0.196 (0.051–0.756)	**0.01**^+^	24 (85.7)	127 (94.1)	2.376	2.646 (0.738–9.488)	0.12
Comprehend the moral and ethical issues in health care	149 (91.4)	88 (89.8)	61 (93.8)	0.817	1.733 (0.520–5.780)	0.376	24 (85.7)	125 (92.6)	1.397	2.083 (0.603–7.193)	0.24
***Ability to see consequences***											
Think about the future	145 (89.0)	89 (90.8)	56 (86.2)	0.865	0.629 (0.236–1.681)	0.35	24 (85.7)	121 (89.6)	0.362	1.440 (0.436–4.756)	0.55
***Critical thinking skills***											
Think critically	142 (87.1)	87 (88.8)	55 (84.6)	0.603	0.695 (0.277–1.746)	0.44	24 (85.7)	118 (87.4)	0.059	1.157 (0.358–3.743)	0.81
***Ability to identify social issues***											
Identify social issues and concerns	151 (92.6)	94 (95.9)	57 (87.7)	3.878	0.303 (0.087–1.052)	0.049	24 (85.7)	127 (94.1)	2.376	2.646 (0.738–9.488)	0.12
***Action skills***											
Take action	148 (90.8)	90 (91.8)	58 (89.2)	0.318	0.737 (0.253–2.140)	0.57	24 (85.7)	124 (91.9)	1.045	1.879 (0.552–6.396)	0.31
Build confidence & take on new responsibilities	147 (90.2)	91 (92.9)	56 (86.2)	1.984	0.479 (0.169–1.357)	0.16	24 (85.7)	123 (91.1)	0.763	1.708 (0.508–5.747)	0.38
***Gaining of knowledge***											
Appreciate and identify gaps or deficiencies in the healthcare system	151 (92.6)	93 (94.9)	58 (89.2)	1.84	0.445 (0.135–1.470)	0.18	22 (78.6)	129 (95.6)	9.809	5.864 (1.734–19.833)	0.002*
Appreciate my own health, living condition	158 (96.9)	97 (99.0)	61 (93.8)	3.464	0.157 (0.017–1.440)	0.063	27 (96.4)	131 (97.0)	0.029	1.213 (0.130–11.282)	0.87
Improve my general knowledge about healthcare	153 (93.9)	92 (93.9)	61 (93.8)	0.001	0.995 (0.269–3.671)	0.99	25 (89.3)	128 (94.8)	1.231	2.194 (0.531–9.067)	0.27
Enhance my understanding of the use of public health measures in resource poor setting	147 (90.2)	92 (93.9)	55 (84.6)	3.787	0.359 (0.124–1.041)	0.052	22 (78.6)	125 (92.6)	5.15	3.409 (1.125–10.333)	0.023*
***Application of knowledge***											
Improve my caregiving skills (e.g. blood pressure measurement, capillary blood glucose measurement)	149 (91.4)	90 (91.8)	59 (90.8)	0.057	0.874 (0.289–2.648)	0.81	24 (85.7)	125 (92.6)	1.397	2.083 (0.603–7.193)	0.24
Apply what I learnt in the training sessions (the one organized before the start of the home visits)	138 (84.7)	80 (81.6)	58 (89.2)	1.737	1.864 (0.731–4.754)	0.19	22 (78.6)	116 (85.9)	0.966	1.665 (0.598–4.640)	0.33

+ Significant when adjusted for age

*Significant when adjusted for gender

### Program feedback

#### Healthcare undergraduates

Majority of the healthcare undergraduates felt more prepared for their practice as healthcare professionals in the future. 92.4% are now more aware of the problems faced by the older persons. 91.9% would recommend the program to their friends (Table 9).

**Table 9:** Quantitative feedback

	% of healthcare undergraduates who agreed (*n* = 185 unless otherwise stated) (95% CI)	% of students who agreed (*n* = 172) (95% CI)
The multi-disciplinary meetings were useful for learning	72.8 (65.7–79.9) (*n* = 169)	
I am more prepared for my practice as a healthcare professional in the future.	80.5 (74.6–85.9)	
I am inspired and empowered to start something new to fulfill a social need	75.1 (68.1–80.5)	
I have achieved my personal goals set at the start of the cycle	76.2 (69.7–82.2)	
I better appreciate the importance of inter-professional collaboration in the care of patients	91.6 (86.3–96.8) (*n* = 95)	
I am now more confident with communicating with the elderly.		62.2 (55.2–69.8)
The lessons learnt during the home visits are applicable to me and my family		75.0 (68.0–81.4)
I will bring back the lessons learnt and educate my family members regarding the importance of healthy lifestyle and health screening		64.0 (56.4–71.5)
The curriculum is useful for my learning		64.0 (57.0–71.5)
I am now more confident in providing basic caregiving skills	90.3 (85.9–94.1)	65.7 (58.7–72.7)
I am now more aware of the problems faced by the elderly	92.4 (88.6–96.2)	83.1 (77.9–88.4)
I would recommend TriGen to my friends	91.9 (87.6–95.7)	91.3 (86.6–95.3)

#### Secondary school students

83.1% of SS students are more aware of problems faced by the older persons and 91.3% would recommend the program to their friends (Table 9)

### Patients’ reported satisfaction and clinical outcomes

Table 10 describes the demographic of our patients There were 116 patients who participated in the program. The mean age is 73.5 years-old. The mean age-adjusted Charlson co-morbidity index is 9.1.

**Table 10:** Patient demographics

Demographic	Variables	Numbers (Percentages)
Age	Mean 73.5 (54–95 years)	
Gender	Males	58 (54.7)
	Female	48 (45.3)
Race	Chinese	71 (67.0)
	Malay	6 (5.7)
	Indian	26 (24.5)
	Others	3 (2.8)
Charlson Comorbidities Index	Mean 9.1 (SD 2.9)	
Lawton IADL	Mean 4.4 (SD 2.4)	
Alone	Yes	12 (19.0)
	No	51 (81.0)
Housing	1-room	13 (21.0)
	2-room	4 (6.5)
	3-room	22 (35.5)
	4-room	18 (29.0)
	5-room	4 (6.5)
	Others	1 (1.6)
Financial	Yes	40 (39.2)
	No	62 (60.8)
Carer	Self	27 (42.9)
	Spouse	8 (12.7)
	Children	26 (41.3)
	Grandchildren	1 (1.6)
	Siblings	1 (1.6)

A Wilcoxon signed-rank test showed a statistically significant decrease in hospital admission rates during the 6 months period before the program and the 6 months period after from a median of 1 visit (range of 0 to 5) to 0 visit (range of 0 to 10). (Z = 2.72, *p* = 0.006). A Wilcoxon signed-rank test showed a statistically significant decrease in emergency department visits during the 6 months period before the program and 6 months period after from a median of 1 visit (range of 0 to 10) to 1 visit (range of 0 to 10). (Z = 2.91, *p* = 0.004).

A total of 51 patients answered the patient feedback survey. The majority (> 80%) felt less lonely and happier because of the home visits. Most (> 50%) felt that they have changed their lifestyle for the better and feel more confident taking care of their own health as a result of the home visit (Table 11).

**Table 11:** Patient feedback

Because of the home visits, I ..	Agree (% of respondents)	Neutral	Disagree	Did not answer (% of all patients)
I understand more about my health problems because of the home visits	35 (71.4)	8 (16.3)	6 (12.2)	57 (53.8)
I feel more confident in taking care of my own health because of the home visits	34 (68.0)	12 (24.0)	4 (8.0)	56 (52.8)
I have changed my lifestyle (e.g. diet, exercise, leaving the house more often, etc.)	25 (50.0)	13 (26.0)	12 (24.0)	56 (52.8)
I want to continue to improve my health because of the home visits	30 (61.2)	11 (22.4)	8 (16.3)	57 (53.8)
I feel less afraid to ask questions about my health because of the home visits	26 (53.1)	12 (24.5)	11 (22.4)	57 (53.8)
I enjoyed the activities done during the home visits	48 (96.0)	2 (4.0)	0 (0.0)	56 (52.8)
I feel less lonely because of the home visits	42 (84.0)	6 (12.0)	2 (4.0)	56 (52.8)
I feel happier because of the home visits	48 (94.1)	3 (5.9)		55 (51.9)
I look forward to the home visits	44 (86.3)	6 (11.8)	1 (2.0)	55 (51.9)
I would like to continue the home visits with a different group of students	32 (65.3)	10 (20.4)	7 (14.3)	57 (53.8)
The students were respectful	49 (96.1)	2 (3.9)	0 (0.0)	55 (51.9)
I found it easy to talk to the students	42 (82.4)	8 (15.7)	1 (2.0)	55 (51.9)
I made friends with the students	37 (72.5)	12 (23.5)	2 (3.9)	55 (51.9)
My family/caregivers enjoy the home visits	26 (70.3)	10 (27.0)	1 (2.7)	69 (65.1)
My family/caregivers are engaged by the team leaders and students during the home visits	23 (69.7)	9 (27.3)	1 (3.0)	73 (68.9)
It was easy to schedule each home visit	40 (78.4)	8 (15.7)	3 (5.9)	55 (51.9)
	**Just nice**	**Too short / Too infrequent**	**Too long / Too frequent**	**Missing**
The duration of home visits is	45 (88.2)	5 (9.8)	1 (2.0)	55 (51.9)
The frequent of the home visits is	43 (86.0)	7 (14.0)	0 (0.0)	56 (52.8)
The number of students per visit is	51 (100.0)	0 (0.0)	0 (0.0)	55 (51.9)

## Discussion

### Reducing ageism and improving knowledge

To our knowledge, this is the first paper that discusses a longitudinal student-initiated, inter-generational, inter-professional home visit program which enables healthcare undergraduates and lay students (SS students) to provide holistic care to the older persons in the community through a service-learning approach.

TriGen decreased ageist attitudes amongst both healthcare undergraduates and SS students, with significant increases in KOP scores for both groups. This effect is greater amongst the healthcare undergraduates, possibly because of their greater involvement in the care of the older persons compared to the SS students. This increase in KOP scores were found in all subgroups of participants (Tables 3 and 4).

TriGen also increased knowledge about the older persons amongst SS students (mean increase of 0.8 ± 0.5, *p*-value = 0.005), but not healthcare undergraduates.

Levy et al. proposed a theoretical model to reduce ageism which consisted of education and positive contact with the older persons [46]. We propose that our program supports the validity of this theoretical model.

Firstly, our program employed education on aging to dispel negative and inaccurate images of older adulthood. There was a correlation between change in KOP scores and PFAQ scores amongst the SS students suggesting that an increase in knowledge of the older persons may reduce ageism. While there was no statistical increase in the PFAQ score for healthcare undergraduates, there was a significant correlation between change in KOP score and change in PFAQ score. (*r* = 0.266, *p* <  0.001). Our qualitative data showed that both healthcare undergraduates and SS students demonstrated greater understanding of the older person. These qualitative data will be published in a separate paper. This is supported by studies that demonstrated a more accurate knowledge of aging is associated with less ageist attitudes [47]. Moreover, educational efforts to increase knowledge of the aging do result in a reduction in ageist attitudes [48, 49].

Secondly, TriGen provided positive contact with the older persons. This is based on the intergroup contact hypothesis which proposes that negative intergroup attitudes stem in part from lack of positive contact between group members [50, 51]. In the qualitative analysis, the healthcare undergraduates and SS students reported that they had a very meaningful experience with the older persons and many enjoyed the intergenerational bonding. Amongst SS students, those who spent more time interacting with the elderly in TriGen had a greater decrease in ageist attitudes, supporting the contact theory.

Lastly, TriGen reduced ageism because it developed empathy and understanding of person-centric care amongst our participants. Most of our participants (more than 90%) reported gain in the skill of being able to think of others and being tolerant of different people (Tables 7 and 8). Moreover, in our qualitative data amongst the healthcare undergraduates, a major theme is that healthcare undergraduates learned to see the patient beyond their diseases and conditions to see them as individuals with unique life stories; they also reported developing empathy. Amongst SS students, a major theme is the development of empathy.

The healthcare undergraduates have a higher pre-intervention mean KOP score (133.0 ± 2.9) compared to the SS students (127.4 ± 1.8), perhaps reflecting the tendency for less ageist individuals to join the healthcare professions and the greater amount of geriatric education and experiences working with older persons the healthcare undergraduates get. The healthcare undergraduates in this study had similar positive attitudes towards the older person as compared to Year 1 and Year 3 medical students in a study by Cheong et al (mean KOP score was 135.2 ± 14.9 and 138.2 ± 13.5 for the Year 1 and Year 3 medical students respectively) [52].

When compared with the junior doctors comprising House Officers, Medical Officers and Registrar in a tertiary hospital described in a previous study done by Lui et al.*,* our healthcare undergraduates had a much higher pre-intervention KOP score (133.0 ± 2.9) [53]. The mean KOP score of the doctors in that study is 114.4 ± 9.0. Empathy levels are known to decrease as training progresses amongst medical students and residents [26–29, 54]. There is possibly such a similar trend in ageism [25]. However, current data conflicts with one study demonstrating a possible increase in ageism as training progresses amongst junior doctors, and another study reporting a possible decrease in ageism with increasing years of seniority amongst medical students. In both studies, the trend was not statistically significant [52, 53]. However, the study population may not be comparable as medical students are different from junior doctors, in that junior doctors’ attitudes could be modified by actual acute hospital practice and negative care experiences with frail older patients.

We would expect that those who live with their grandparents and/or who had previous experiences volunteering with the older persons would have higher pre-intervention mean KOP scores. Indeed, healthcare undergraduates who live with their grandparents had higher mean pre-intervention KOP scores. However, this is not seen amongst SS students. Interestingly, previous experiences volunteering with older persons were not associated with significant differences in mean pre-intervention KOP scores amongst both healthcare undergraduates and SS students; reasons why deserve to be explored in future studies.

### Personal development and gaining of skills

Both healthcare undergraduates and SS students found that TriGen was effective in increasing their understanding of all 9 domains of the FIPSE. This is likely because TriGen is a student-initiated project with high levels of student involvement in all phases of the project from conceptualization, and planning to implementation. Moreover, the longitudinal nature of TriGen conducted over 6 months provided for ample opportunities to develop skills and inculcate values. There were some differences in the perceived educational value for students of differing gender, age and clinical experience, but it is known that these can affect students’ attitudes towards learning [55–57]. In contrast to reports by various authors in the Asian and Western setting that healthcare students with no prior clinical exposure are more likely to have higher perceived educational value [42, 58, 59], we found that the healthcare undergraduates with clinical experience gained more in being able to apply what they have learned in the training sessions to the home visits and improved clinical diagnostic skills. We postulate that this is because TriGen is a longitudinal program involving patients with complex care needs who have frequent admissions. Hence, healthcare undergraduates with more clinical experience were more likely to be able to grasp and process the complexities.

Healthcare undergraduates were more likely to perceive TriGen to have helped them improve their leadership skills. Approximately 10% more healthcare undergraduates as compared to SS students agreed to the statement “TriGen has helped me to improve my leadership skills.” This is likely due to the nature of the involvement of both groups in TriGen: the healthcare undergraduates, as the leaders of each visitation team, were directly accountable to the community nurses and other hospital healthcare professionals in KTPH, and took charge of representing the patient’s interests in multi-disciplinary meetings. The SS students’ role were relatively more passive, and they were mostly led by the healthcare undergraduates in interacting with the older persons in home visits. As such, they had a much smaller degree of ownership and responsibility in the care of the older persons.

### Impact on the patients

Patients were observed to have a reduction in the number of hospital admissions and emergency department visits after participating in TriGen. The evidence suggests that integrated geriatrics care provided through home visits may reduce acute hospital use [4–6]. Our study suggests that a student-initiated, intergenerational, interprofessional program supervised by healthcare professionals can potentially achieve similar efficacy as a home visit program run by a professional geriatrics unit. Most patients reported that they have made positive changes to their lifestyles and are more confident of self-care as a result of the program. Also, another possible explanation for the reduction in utilization of healthcare resources could be secondary to the improved psychological and social wellbeing [60]. Many reported that they enjoyed the home visits and felt less lonely and happier as a result of the program.

### Limitations

Our study has several limitations. First, as participation for both undergraduates and SS students was voluntary, the participants were likely to be self-selected and this would reduce the generalizability of our findings. However, it is noted that the mean pre-intervention KOP score is similar to that reported in other cohorts of medical students and nursing students. Second, the learning outcomes were self-reported. Third, we have no longitudinal data on the long-term learning outcomes. Fourth, the healthcare resources utilization data are not controlled and the reduction in utilization of healthcare resources cannot be conclusively attributed to the program. Lastly, despite PFAQ being previously validated, our study revealed a low Cochrane’s alpha for the instrument. The results should be interpreted with caution.

## Conclusion

In summary, TriGen demonstrates the potential of a student-initiated, longitudinal, inter-generational and inter-professional home visit program to reduce ageism, develop soft skills, inculcate values amongst SS students and healthcare undergraduates. In addition, TriGen potentially reduces hospital admissions and emergency department visits, and loneliness amongst frequently admitted older patients. We hope that our experience will encourage other institutions and communities to adopt this concept that we have shown to be feasible and impactful.

## Data Availability

The datasets used and/or analysed during the current study are available from the corresponding author on reasonable request.

## Abbreviations

AIP program: Aging-in-place program
ANOVA: Analysis of variance
CI: Confidence Interval
FIPSE: Fund for the Improvement of Post-Secondary Education Survey Instrument
KOP: Kogan Attitude towards Old People Scale
KTPH: Khoo Teck Puat Hospital
NUS YLLSoM: National University of Singapore Yong Loo Lin School of Medicine
NWCDC: North West Community Development Council
PFAQ: Palmore Facts on Aging Quiz
SPSS: Statistical Package for Social Sciences
SS students: Secondary School students
TriGen: Tri-Generationsl HomeCare
